# Editorial: Epigenetic Mechanisms and Their Involvement in Rare Diseases

**DOI:** 10.3389/fgene.2021.755076

**Published:** 2021-09-01

**Authors:** Mojgan Rastegar, Dag H. Yasui

**Affiliations:** ^1^Department of Biochemistry and Medical Genetics, Max Rady College of Medicine, Rady Faculty of Health Sciences, University of Manitoba, Winnipeg, MB, Canada; ^2^Department of Medical Microbiology and Immunology, University of California Davis School of Medicine, Davis, CA, United States

**Keywords:** epigenetics and rare diseases, MeCP2 isoforms and rett syndrome (RTT), DNA methylation and histone modifications, ATRX and gene regulatory mechanisms, activity dependent neuroprotective protein (ADNP) and chromatin remodeling, Beckwith-Wiedemann Syndrome (BWS) and Prader-Willi Syndrome (PWS), O-linked-D-N-acetylglucosamine (O-GlcNAc), MYCN-related epigenetic factors and non-coding regulatory RNAs

Epigenetic mechanisms are diverse modes of gene regulation, acting independent of genetic sequences. Epigenetics involves an array of “readers,” “writers,” and “erasers,” with key roles in development, health, and disease. One important aspect of epigenetics is involvement in rare diseases. This special topic covers a series of original research and review articles that further our knowledge about epigenetic mechanisms in rare diseases.

One well-studied example of rare diseases caused by genetic mutations is Rett Syndrome (RTT). RTT is due to *de novo* mutations in the X-linked Methyl CpG Binding Protein 2 (*MECP2*) gene. The multi-functional “MeCP2” protein plays important roles in neuronal maturation and brain development.

Focusing on RTT, Sharifi and Yasui, provide an overview about MeCP2 protein biology and its functional relevance to RTT. The authors explain how *MECP2* mutations contribute to disease mechanisms, describing lessons learnt from RTT mice and model systems. They describe how *MECP2* expression is distributed among different organs, using helpful schematics. They further discuss MeCP2 DNA binding activities, and its association not only with CpG dinucleotide methylation, but also with CpH methylation in the context of CpA, CpC, or CpT. The authors elaborate on MeCP2 function as a dual transcriptional regulator, as an activator or effective suppressor of gene transcription. The authors also discuss MeCP2 splice variants; MeCP2E1 and MeCP2E2, MeCP2 role in liquid phase separation, and potential therapeutic strategies for RTT.

Complementing the first paper, Good et al., offer a timely review entitled “MeCP2: the genetic driver of Rett Syndrome epigenetics.” The authors discuss how RTT-associated *MECP2* gene mutations can modify its DNA binding activities, and chromatin bundling capabilities, while altering MeCP2 protein stability. They further discuss the role of MeCP2 in alternative splicing and micro-RNA processing. Other aspects of MeCP2 function, diverse protein domains, and different mutations are also well-discussed. Interestingly, the authors explain the impact of proteasomal degradation through MeCP2 PEST sequences, and circadian-dependent dynamics of MeCP2 isoforms. Finally, the authors highlight the complexity of RTT pathology with differential relevance of MeCP2 isoforms.

The third paper on RTT is an original research article by Pejhan et al. The authors studied MeCP2 homeostasis regulatory network in the frontal cerebrum, hippocampus, amygdala, and cerebellum of post-mortem brain tissues from RTT patients and non-RTT controls. The authors establish that in humans, the MeCP2 homeostasis network (MeCP2E1/E2-BDNF-*miR132*) is brain-region specific. Their correlational studies suggested that cerebellum is the one tested brain region that may involve all these regulatory components. Their findings highlighted that MeCP2 isoform-specific protein levels do not fully follow their corresponding transcript levels. The authors further show that components of this regulatory network are significantly compromised in all tested brain regions, with reduced levels in RTT patients, except for *miR132* in the cerebellum. Among the two complementary *miR132-3p* and *miR132-5p* strands, the former appeared as the dominant *miR132* in these brain regions. This study is among limited research studies to examine human brain tissues for this rare disease.

In the section “Epigenetics in Prader-Willi syndrome,” Mendiola and LaSalle present the emerging evidence of how loss of the small, non-coding RNA, *SNORD116*, contributes to neurological defects. The authors present evidence supporting Prader-Willi syndrome (PWS) as a metabolic disorder. They explain that patients present with poor feeding behavior as neonates, then progress to extreme hunger and obesity by early childhood. PWS is an imprinting disorder as *SNORD116* in neurons is expressed solely by the paternal allele. Loss of *SNORD116* expression by deletion, uniparental disomy, or abnormal DNA methylation of the imprinting control region commonly causes the phenotype. What is intriguing is the evolution of *SNORD116* function for sleep regulation and crosstalk with other key imprinted genes.

Similar to Prader-Willi Syndrome, Beckwith-Wiedemann Syndrome (BWS) is a rare imprinting disorder as paternally expressed *IGF2* and maternally expressed *H19* are altered. In their review, Papulino et al. present the complexity of the *IGF2/H19/CDKN1C* locus and how epigenetic DNA methylation regulates gene expression. As *IGF2* encodes a growth factor and *CDKN1C* encodes a cell cycle regulator, their emphasis is on examining the link with organ overgrowth and elevated cancer risk in BWS patients. In addition, valuable information is provided on the potential of repurposed “epi drugs” for treatment of BWS.

In contrast to DNA methylation and non-coding RNA, epigenetic defects in PWS and BWS, Fallah et al. describe histone methylation and acetylation defects underlying selected neurodevelopmental disorders such as Kabuki syndrome and Rubestein-Taybi syndrome, respectively. One provided key piece of information is the comparison of the broad range of tissues affected by these disorders. Of particular interest is the correlation between intellectual disability and dysmorphic body features. Although this article focuses on basic epigenetic mechanisms, treatments targeting histone defects underlying these disorders are now in use in clinics for patient treatments.

In a review by Timpano and Picketts, defective chromatin remodeling is discussed in the context of abnormal gene expression, and impacts on fundamental cellular activities, such as DNA replication, DNA repair, cellular proliferation and differentiation. Members of ATP-dependent chromatin remodeling factors include SWI/SNF, ISWI, INO80, CHD, and ATRX. One example of their association with rare disorder is the ATR-X Syndrome, a congenital disorder in males. These patients display heterogeneous phenotypes, although the disease is caused by single *ATRX* gene mutation(s). The authors provide an overview of ATRX interacting partners and biochemical functions. Further discussions include phenotypic and behavioral outcomes in different model systems of disease, transgenic mice, and potential ATRX roles in forebrain development.

Sun et al. study an epigenetic disorder in their original research. Activity Dependent Neuroprotective Protein (ADNP) is associated with a recently described disease; Helsmoortel-Van der Aa syndrome. The authors identify ANDP physical interaction with chromatin remodeling factors BRG1 and CHD4 and co-occupancy of these factors at intergenic sites. Interestingly, 75% of differentially expressed genes (DEG) from deletion or reduction in *Adnp* expression in embryonic stem cells (ESC) were upregulated. Genome wide chromatin accessibility was elevated along with altered histone marks due to ADNP loss, suggesting regulation of developmental genes through chromatin compaction. A connection between Helsmoortel-Van der Aa syndrome and other neurodevelopmental disorders such as Prader-Willi and Rubenstein-Taybi syndromes was suggested by common symptoms of intellectual disability and dysmorphic features.

The review by Konzman et al. establishes that O-GlcNAc transferase (OGT) enzyme regulation of post-translational modification O-linked-D-N-acetylglucosamine (O-GlcNAc) is involved in key cellular processes. The authors provide detailed examples of how environmental signals integrate into epigenetic DNA regulation of the human nervous system. The insights into OGT/O-GlcNAc function in human neurologic processes were obtained by studies of OGA/OGT function in model organisms such as *Drosophila* over the past several years.

Another original research on neuroblastoma is contributed by Krstic et al. Indeed, neuroblastoma is a rare disease, although it constitutes a high fraction of cancer deaths in children. The authors applied a chemo-genomic approach to study the global MYCN-epigenetic interactions. Among many epigenetic factors that they identified as MYCN targets, there are HDAC2, CBP, and CBX8. The authors reported that expression level of most MYCN-related epigenetic factors was associated with predictive patient outcomes. They completed a compound library screen for epigenetic proteins that showed wide response of neuroblastoma cells to epigenetic modulators. A particular chemical was C646, and the identified susceptibility of these cells correlated with MYCN expression.

Chen et al., provide a review entitled “A comprehensive genomic analysis constructs miRNA-mRNA interaction network in hepatoblastoma.” While hepatoblastoma is rare, it is considered as the most common type of hepatic tumor in pediatric patients. By comparing hepatoblastoma and normal liver tissues, the authors identified 580 differentially expressed upregulated transcripts, and 790 downregulated transcripts. Among these, they noticed differentially expressed miRNAs. Analysis of protein-protein interaction network was further completed. The authors concluded that certain microRNAs, transcription factors, and hub genes act as potential regulators in hepatoblastoma.

## Author Contributions

All authors listed have made a substantial, direct and intellectual contribution to the work, and approved it for publication.

## Conflict of Interest

The authors declare that the research was conducted in the absence of any commercial or financial relationships that could be construed as a potential conflict of interest.

## Publisher's Note

All claims expressed in this article are solely those of the authors and do not necessarily represent those of their affiliated organizations, or those of the publisher, the editors and the reviewers. Any product that may be evaluated in this article, or claim that may be made by its manufacturer, is not guaranteed or endorsed by the publisher.

